# Defining Immune Engagement Thresholds for *In Vivo* Control of Virus-Driven Lymphoproliferation

**DOI:** 10.1371/journal.ppat.1004220

**Published:** 2014-06-26

**Authors:** Cristina Godinho-Silva, Sofia Marques, Diana Fontinha, Henrique Veiga-Fernandes, Philip G. Stevenson, J. Pedro Simas

**Affiliations:** 1 Instituto de Medicina Molecular, Faculdade de Medicina, Universidade de Lisboa, Lisboa, Portugal; 2 Sir Albert Sakzewski Virus Research Center and Queensland and Children's Medical Research Institute, University of Queensland, Brisbane, Queensland, Australia; Dartmouth Medical School, United States of America

## Abstract

Persistent infections are subject to constant surveillance by CD8^+^ cytotoxic T cells (CTL). Their control should therefore depend on MHC class I-restricted epitope presentation. Many epitopes are described for γ-herpesviruses and form a basis for prospective immunotherapies and vaccines. However the quantitative requirements of *in vivo* immune control for epitope presentation and recognition remain poorly defined. We used Murid Herpesvirus-4 (MuHV-4) to determine for a latently expressed viral epitope how MHC class-I binding and CTL functional avidity impact on host colonization. Tracking MuHV-4 recombinants that differed only in epitope presentation, we found little latitude for sub-optimal MHC class I binding before immune control failed. By contrast, control remained effective across a wide range of T cell functional avidities. Thus, we could define critical engagement thresholds for the *in vivo* immune control of virus-driven B cell proliferation.

## Author Summary

Chronic viral infections cause huge morbidity and mortality worldwide. γ-herpesviruses provide an example relevant to all human demographics, causing, *inter alia*, Hodgkin's disease, Burkitt's lymphoma, Kaposi's Sarcoma, and nasopharyngeal carcinoma. The proliferation of latently infected B cells and their control by CD8^+^ T cells are central to pathogenesis. Although many viral T cell targets have been identified *in vitro*, the functional impact of their engagement *in vivo* remains ill-defined. With the well-established Murid Herpesvirus-4 infection model, we used a range of recombinant viruses to define functional thresholds for the engagement of a latently expressed viral epitope. These data advance significantly our understanding of how the immune system must function to control γ-herpesvirus infection, with implications for vaccination and anti-cancer immunotherapy.

## Introduction

The gamma-herpesviruses (γHVs) infect >90% of humans and cause diseases including nasopharyngeal carcinoma, African Burkitt's lymphoma and Kaposi's Sarcoma. Their colonization of circulating memory B cells is crucial to persistence and hence to disease ontogeny. Viral latency gene expression in B cells provides an immune target [1] that has been exploited to prevent lymphoproliferative disease in acutely immunodeficient patients by T cell transfer [2]. However, extending this approach to established cancers and developing related vaccines have proved difficult. A significant problem is that the narrow species tropisms of human γHVs severely restrict *in vivo* analysis, and hence an understanding of how empirical therapies such as adoptive T cell transfer work.

Immune recognition can be assayed *in vitro*; but while Epstein-Barr virus (EBV) latency gene products drive autonomous B cell proliferation *in vitro*, most *in vivo* infected cells are resting memory B cells that have passed though lymphoid germinal centers (GCs) [3]. This makes difficult *in vitro* analysis of *in vivo* immune control. One way to make progress is to study related viruses that are experimentally more accessible. Probably the best characterized is Murid Herpesvirus-4 (MuHV-4, archetypal strain MHV-68) [4]–[6]. MuHV-4 is more closely related to the Kaposi's Sarcoma-associated Herpesvirus (KSHV) than to EBV [7]. However it shares many features of host colonization with EBV, for example it exploits lymphoid GCs to establish persistence in circulating memory B cells [8]–[10]. Therefore it can be used to reveal fundamental mechanisms of γHV/host interaction.

MuHV-4 studies have shown that γHV-driven lymphoproliferation occurs in complex lesions incorporating T cell evasion and infected cells with distinct patterns of viral gene expression [10]. In addition to cis-acting T cell evasion during episome maintenance [11], [12], EBV inhibits the transporter associated with antigen processing (TAP) via BNLF2a [13]–[15] and MHC class I export to the cell surface via BILF1 [16], [17]; KSHV degrades MHC class I and other immune receptors via K3 and K5 [18]; and MuHV-4 degrades MHC class I and TAP via MK3 [19]–[21]. Disrupting MK3 impairs virus-driven lymphoproliferation [22].

The γHVs also evade immune recognition during latency by expressing few CTL targets. However a gene that modulates signaling through the B cell receptor - M2 in MuHV-4 [23]–[26], LMP-2A in EBV [27] and K1 in KSHV [28] - is expressed more widely than growth program genes [3], and shows protein sequence diversity [29]–[33] consistent with immune selection. More directly, the presence of an H2K^d^ binding epitope in M2 [34], [35] significantly reduces long-term MuHV-4 latent loads in BALB/c mice [29]. Therefore despite viral evasion, CTL help to regulate long-term infection [36], [37], and CTL recognition of M2/K1/LMP-2A, which in EBV may extend also to EBNA3A/B/C [38], [39], provides a potential point of attack. LMP-2A is also a candidate vaccine target for nasopharyngeal carcinoma [40]. Thus, how M2/K1/LMP-2A recognition works *in vivo* is important to understand.

CTL effector capacity broadly correlates with functional avidity, as determined by the capacity of T cell receptor (TcR) engagement to trigger CTL proliferation, cytokine production and target cell lysis at limiting antigen dose [41]. Therefore with limited γHV protein expression during latency, peptide affinity for MHC class I and TcR functional avidity are likely to be crucial for immune control. The diversity of LMP-2A, K1 and M2 prompted us to analyze *in vivo* the consequences of varying MHC class I binding and TcR functional avidity for a single epitope derived from M2. These parameters affected dramatically the control of virus-driven lymphoproliferation, even in the context of immune evasion. The capacity of MuHV-4 to correlate biochemical interactions with *in vivo* immune function allowed us to establish quantitative guidelines for infection control.

## Results

### Characterization of altered peptide ligands (APLs) by MHC class I binding and TcR functional avidity

**Figure 1:**
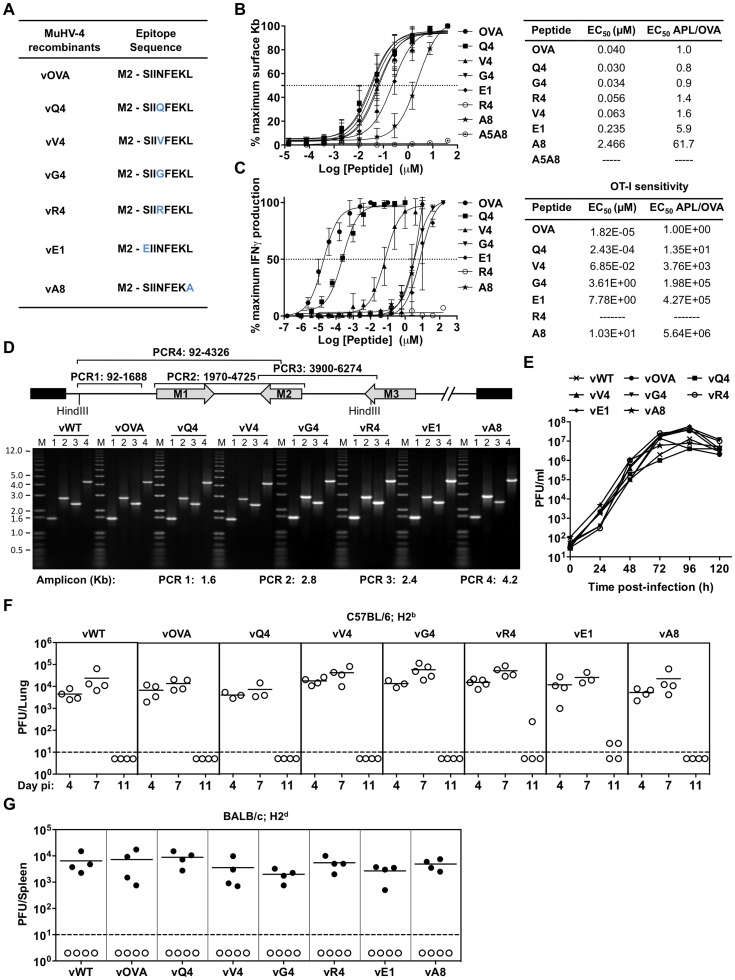
Characterization of APLs by MHC class I binding and TcR functional avidity, and generation of MuHV-4 recombinants expressing OVA or APLs linked to M2. (A) Amino acid sequences used to generate MuHV-4 recombinants. Blue residues denote amino acid alterations introduced into native OVA. (B) Capacity of OVA and APL peptides to stabilize H2K^b^ on TAP deficient RMA/S cells. Half-maximum effective concentration (EC_50_) values were calculated from dose-response curves. The experiment was repeated 3 times. (C) Functional avidities of OT-I CTL for OVA and APL peptides were determined by IFNγ production. EC_50_ and APL/OVA EC_50_ ratios are shown. This experiment was repeated in duplicates 4 times (D) PCR analysis of recombinant viral DNA to confirm genome integrity in the *Hin*DIII-E region, with schematic representation of the MuHV-4 genome, amplicon genomic co-ordinates and predicted PCR product sizes. (E) Multi-step growth curves of viruses in BHK-21 (0.01 PFU/cell). Virus titres were determined by plaque assay. *In vitro* lytic replication kinetics of the recombinant viruses were not significant different from vWT (p>0.05, by ordinary one-way ANOVA followed by Dunnett's multiple comparisons test). (F) Virus replication in lungs of i.n. infected C57BL/6 (H2^b^) mice was quantified by plaque assay. No MuHV-4 recombinant showed a deficit relative to vWT (p>0.05, using ordinary one-way ANOVA followed by Tukey's multiple comparisons test). (G) Latent infection in spleens of BALB/c (H2^d^) mice was determined by explant co-culture assay (closed symbols) at 14 days post-infection. Pre-formed infectious virus were measured by plaque assay (open symbols). Latent loads of MuHV-4 recombinants expressing OVA or APLs were not significantly different to vWT (p>0.05, by ordinary one-way ANOVA followed by Dunnett's multiple comparisons test). In panels F and G each point shows the titre of 1 mouse, horizontal lines show arithmetic means and dashed horizontal lines indicate the detection limit of the assay.

To understand the CTL recognition requirements for γHV infection control, we expressed from MuHV-4 a well-characterized, H2K^b^-restricted epitope comprising amino acid residues 257–264 of ovalbumin (OVA), or APL derivatives (Figure 1A). OVA binds to H2K^b^ with high affinity (K_D_ = 4.1 nM) [42]. We compared OVA and APL binding by H2K^b^ stabilization on TAP-deficient RMA/S cells (Figure 1B) [43]. The OVA concentration giving 50% maximal stabilization (EC_50_) was 40 nM, in close agreement with published data [44]. APLs Q4, V4, G4 and R4 were similar to OVA (EC_50_ within 2-fold), consistent with residue 4 being solvent-exposed in the H2K^b^-peptide complex [45]. E1 required 6-fold more peptide for equivalent H2K^b^ stabilization, consistent with this residue being only partly exposed; A8, which has a mutated anchor residue, required 10-fold more peptide again; and the control peptide A5A8, with 2 mutated anchor residues, gave no significant stabilization. The H2K^b^/OVA/β_2_M complex has an estimated half-life of 8 h [44]. Its stability is determined primarily by the peptide off-rate, so the E1 complex is likely to have a half-life of approximately 1.3 h.

We assessed the functional avidity of the H2K^b^-OVA-specific TcR of OT-I [46] for each APL by *ex vivo* stimulation of CD8^+^ T cells from OT-I mice with graded peptide doses (Figure 1C). There was a clear hierarchy in dose-response, with OVA>Q4 (14-fold)>V4 (a further 279-fold)>G4 (53-fold further still), consistent with published data [47]. The R4 antagonist peptide [48], [49] gave no stimulation. As predicted E1 and A8, which have lower MHC class I binding, generated the lowest dose-responses.

### Generation of MuHV-4 recombinants expressing OVA or APLs linked to M2

We next introduced each epitope at the MuHV-4 M2 C-terminus to ensure expression in latency without compromising M2 function [29]. CTL recognition of an endogenous M2 epitope reduces long-term MuHV-4 latent loads in H2^d^ mice [29]. The lack of an endogenous H2^b^-restricted M2 epitope therefore allowed us to introduce new targets in a context where this is known to be important. Each recombinant virus was also made with a yellow fluorescent protein (YFP) reporter construct [50] to aid infection tracking (Figure S1). Correct epitope insertion and assembly of the surrounding genome were demonstrated by PCR of plaque-purified viral DNA (Figure 1D). Each recombinant virus showed equivalent *in vitro* growth (Figure 1E), equivalent lytic replication in the lungs of intranasally (i.n.) infected C57BL/6 mice (Figure 1F) - with peak titers at 4–7 days post-inoculation and clearance by day 11 - and normal latency establishment in H2^d^ BALB/c mice - with equivalent splenic infectious center assay titers 14 days after i.n. inoculation (Figure 1G). Therefore none showed a replication defect independent of H2^b^-restricted latent epitope expression.

### MHC class I binding by a latency-associated epitope impairs host colonization

**Figure 2:**
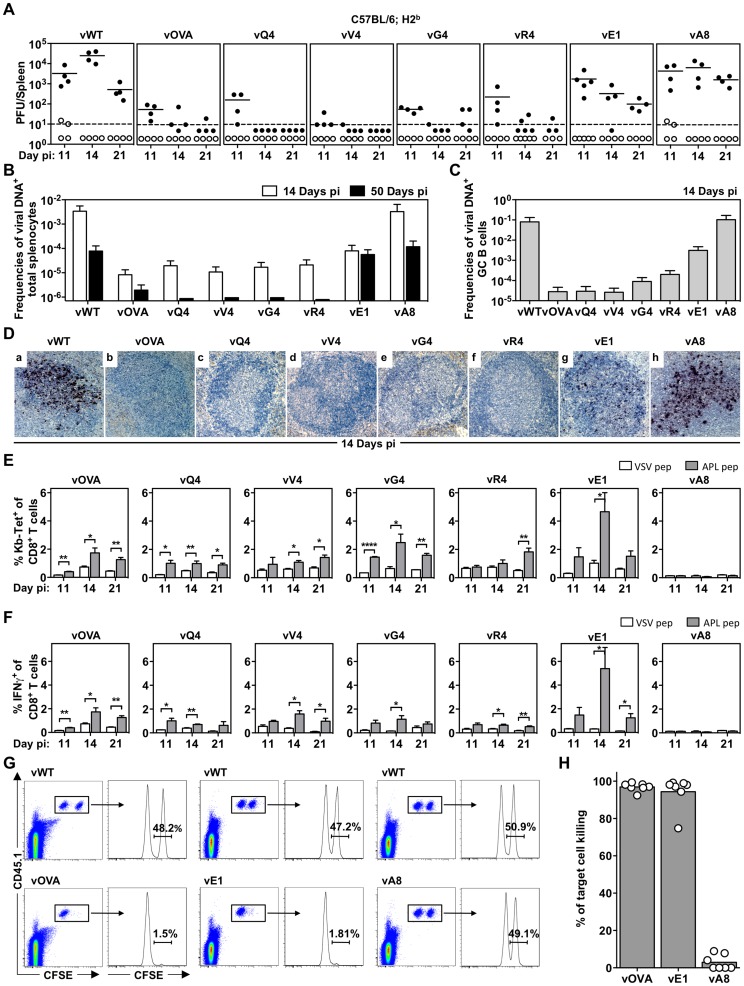
MHC class I binding by a latency-associated epitope impairs host colonization. C57BL/6 mice were infected i.n. with 10^4^ PFU of the indicated viruses. (A) The latent load in spleens was determined by explant co-culture assay (closed symbols) and pre-formed infectious virus was quantified by plaque assay (open symbols). Each point shows the titre of 1 mouse, horizontal lines arithmetic means and dashed horizontal line limit of detection of assay. At day 14, vOVA, vQ4, vV4, vG4, vR4 and vE1 latent loads were significantly below vWT (p<0.05, by two-tailed unpaired t-test). vA8 latency loads were not significantly different from vWT (p = 0.07). (B–C) Reciprocal frequencies of viral DNA^+^ cells in (B) total splenocytes or (C) GC B cells. Bars represent the frequency of viral DNA^+^ cells with 95% confidence intervals. (D) Representative spleen sections showing dark stained latently infected cells by *in situ* hybridization. (E) % tetramer positive CD8^+^ T cells at each time point from spleens (arithmetic mean +/− SEM of 3 independent assays). * p<0.05, ** p<0.01, **** p<0.0001; using a two-tailed unpaired t-test. (F) Functional capacity of splenic CTL determined by intracellular interferon-gamma staining after *ex vivo* stimulation. Data show % CD8^+^ T cells responding to each peptide (arithmetic mean +/− SEM of 3 independent assays). * p<0.05, ** p<0.01; using a two-tailed unpaired t-test. (G–H) *In vivo* CTL activity at 11 days post-infection. (G) At day 10 post-infection 50∶50 mixes consisting of 2×10^6^ unpulsed CD45.1^+^ CFSE^lo^ splenocytes and 2×10^6^ OVA-, E1- or A8-pulsed CD45.1^+^ CFSE^hi^ splenocytes were transferred intravenously into vOVA, vE1 or vA8 infected C57BL/6 mice. The same mix of cells was transferred into vWT infected mice C57BL/6 as internal control. In the next day, the proportion of CFSE^hi^ and CFSE^lo^ cells among CD45.1^+^ cells recovered from the spleen was analysed by FACS. Representative FACS plots showing % of unpulsed CD45.1^+^ CFSE^lo^ and OVA-, E1, or A8-pulsed CD45.1^+^ CFSE^hi^ splenocytes. (H) % target cell killing. Three to four mice were analyzed per group, and experiments repeated three times.

We then tested latency establishment in H2^b^ mice. Infectious center assays (Figure 2A) showed attenuation of any virus with an H2K^b^ binding epitope attached to M2 (vOVA, vQ4, vV4, vG4, vR4): splenic infection was established at day 11, but then cleared rather than amplified by days 14–21. In contrast, the virus expressing a poorly binding epitope (vA8) was indistinguishable from the epitope-negative wild-type (vWT). Interestingly vE1, which expresses an epitope with 6-fold lower EC_50_ for H2K^b^ stabilization (Figure 1B), showed an intermediate phenotype with normal titers at day 11 followed by a gradual reduction.

Not every latently infected cell necessarily reactivates its virus *ex vivo*. We therefore used PCR of viral DNA at limiting dilution (Figure 2B; Table 1) as a second measure of infected cell frequency. We looked at the peak of latent infection (14 days post-inoculation) and at the steady state (50 days). These results supported the infectious centre assays: vOVA, vQ4, vV4, vG4 and vR4 were all markedly attenuated (>100-fold reduction); vA8 was equivalent to vWT; and vE1 showed an intermediate phenotype, with strongly decreased acute titers but long-term titers close to vA8 and vWT. MuHV-4-specific CTL responses peak at 14–21 days post-infection [51]. Thus a weakly binding latent epitope (E1) allowed some control when CTL responses were at their peak, but not in the long-term when CTL responses decrease in size.

**Table 1:** Reciprocal frequency of MuHV-4 infection in total splenocytes^a^ of C57BL/6 mice.

Virus	Day p.i.	Reciprocal frequency^b^ of viral DNA^+^ cells (95% CI)	
vWT	14	296	(179–856)
	50	12,770	(7,900–33,288)
vOVA	14	121,005	(75,230–309,065)
	50	517,114	(316,845–1,405,472)
vQ4	14	51,426	(32,333–125,586)
	50	id	≥1,149,446^c^
vV4	14	92,857	(57,599–239,405)
	50	id	≥1,053,659^c^
vG4	14	59,253	(37,537–140,588)
	50	id	≥1,053,659^c^
vR4	14	47,755	(29,622–123,123)
	50	id	≥1,264,391^c^
vE1	14	12,576	(7,445–40,375)
	50	17,810	(11,400–40,677)
vA8	14	307	(212–962)
	50	8462	(4970–28,436)

a Data were obtained from pools of 4 to 5 spleens.

b Frequencies were calculated by limiting-dilution analysis with 95% confidence intervals (CI).

c Estimated based upon less than 3 different dilution sets.

id; indeterminable.

MuHV-4 colonizes multiple cell types in acutely infected spleens. Many are B cells, which change in phenotype as they pass through germinal centers; others are myeloid cells. The main proliferating population is GC B cells, and these also connect most directly to the long-term latency reservoir of resting memory B cells [9], [10]. Therefore to understand better the relationship between acute and long-term viral loads, we measured viral genome prevalence in flow cytometrically sorted GC B cells (Figure 2C; Table 2). They showed marked reductions for vOVA, vQ4, vV4, vG4 and vR4, equivalent frequencies for vA8 and vWT, and intermediate frequencies for vE1. These data were further supported by *in situ* hybridization for latently expressed viral tRNA/miRNA homologs [29] (Figure 2D), which showed abundant GC infection by vWT and vA8, severely impaired infection by vOVA, vQ4, vV4, vG4 and vR4, and intermediate infection by vE1. Therefore susceptibility to CTL attack during acute lymphoproliferation varied with cell type, and the relative sparing of vE1^+^ GC B cells appeared to allow high long-term viral loads.

**Table 2:** Reciprocal frequency of MuHV-4 infection in GC B cells^a^ of C57BL/6 mice at 14 days post-infection.

Virus	Reciprocal frequency^b^ of viral DNA^+^ cells (95% CI)		% Cells^c^	% Purity^d^
vWT	12	(8–34)	4.63	96.1
vOVA	35,463	(21,819–94,657)	4.06	96.3
vQ4	33,847	(19,882–113,738)	3.63	97.6
vV4	44,687	(23,952–92,597)	4.03	97.4
vG4	11,092	(7,184–24,318)	5.76	96.0
vR4	5,016	(3,268–10,785)	5.66	97.5
vE1	323	(211–687)	4.13	96.5
vA8	10	(6–25)	4.18	96.6

a Data were obtained from pools of 5 spleens.

b Frequencies were calculated by limiting-dilution analysis with 95% confidence intervals (CI).

c The percentage of GC B cells from total spleen was estimated by FACS analysis.

d The purity of sorted cells was determined by FACS analysis.

### CTL responses to epitopes expressed in latent infection

We measured epitope-specific CTL responses with H2K^b^-peptide tetramers (Figure 2E) and by staining for intracellular IFN-γ after *ex vivo* stimulation (Figure 2F). Responses to vA8 were uniformly low despite high viral loads, presumably because this epitope was not produced in sufficient amounts to compensate for its poor H2K^b^ binding. Responses to vOVA, vQ4, vV4, vG4 and vR4 were detectable, although small compared to those reported for lytic antigens [51]. Surprisingly, the largest CTL response was elicited by the intermediate phenotype virus, vE1. This could not be explained by lytic infection, since this was high in lungs for all viruses (Figure 1F).

We confirmed the functionality of vE1-specific CTL by *in vivo* killing of CFSE-labelled, peptide-exposed targets (Figure 2G,H): vE1-induced CTL showed target cell elimination comparable to vOVA, whereas mice infected with vWT or vA8 showed none. Therefore the relatively weak H2K^b^ binding of E1 was sufficient to stimulate large, functional CTL responses, but not for those CTL to curtail efficiently virus-driven lymphoproliferation. This result suggested that at least for vE1, most CTL stimulation comes from a population distinct from that engaged in lymphoproliferation.

### CTL functional avidity also determines infection control by latency epitope recognition

**Figure 3:**
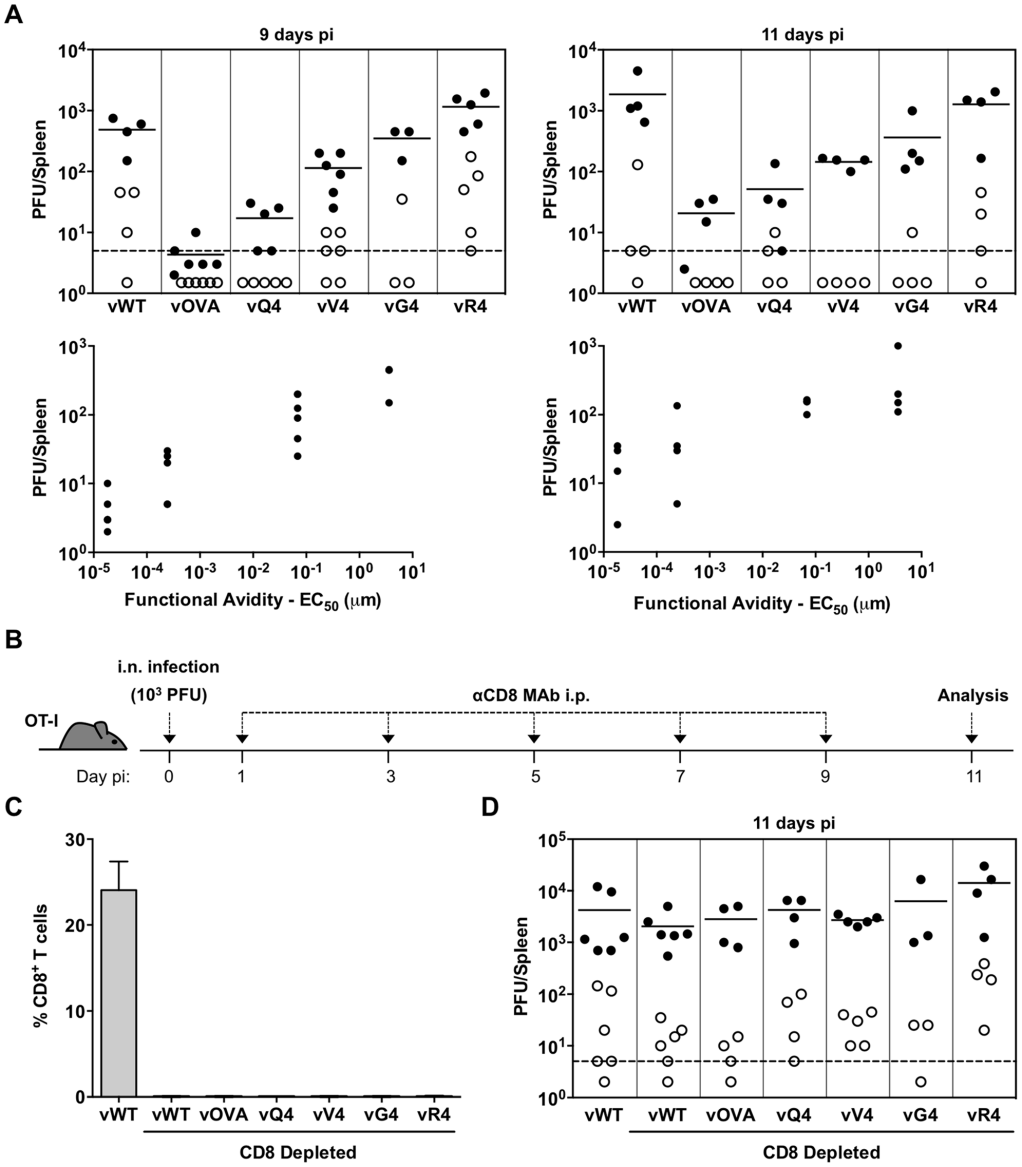
CTL functional avidity also determines infection control by latently expressed epitope recognition. (A) OT-I mice were infected i.n. (10^3^ PFU). Splenocytes were titrated for latent virus by explant co-culture (closed circles) and for pre-formed infectious virus by plaque assay (open circles). At 9 days vOVA, vQ4 and vV4 showed significantly less latent infection compared to vWT (vOVA p = 0.0014, vQ4 p = 0.004, vV4 p = 0.009; by Student's 2-tailed unpaired t-test). vG4 and vR4 latent infections were not significantly different to vWT (vG4 p = 0.46, vR4 p = 0.09). Graphs show the correlation between TcR functional avidity (determined in Figure 1C) and splenic latent load (day 9: p = 0.04, r_s_ = 0.91; day 11 p = 0.05, r_s_ = 0.90; according to Pearson's correlation). (B) CD8 T^+^ cells were depleted from i.n. infected OT-I mice by intraperitoneal injection of anti-CD8 monoclonal antibody (MAb). (B) Schematic diagram of the experimental setting. (C) Data show the percentage of CD8^+^ T cells of total splenocytes (arithmetic mean +/− SEM) in control (non-depleted) and depleted mice. (D) Spleens were titrated for latent (closed circles) and lytic (open circles) infection. Latent loads of the epitope recombinants were not significantly different to vWT latent loads in CD8-depleted mice (p>0.05; ordinary one-way ANOVA followed by Dunnett's multiple comparisons test). Data were reproduced in two independent experiments. Each point shows the titre of 1 mouse, horizontal lines arithmetic means and dashed lines the limit of detection of the assay.

The capacity of C57BL/6 mice to control MuHV-4-driven lymphoproliferation through the recognition of latently expressed OVA, Q4, V4, G4 or R4 indicated that the key requirement in a polyclonal TcR setting is the availability of an epitope capable of strong MHC class I binding: T cells from the naive repertoire could recognize either OVA or an APL. However responses to EBV can involve oligoclonal or even monoclonal CTL expansions [52]–[54]. Therefore to understand better the quantitative requirements of TcR functional avidity for *in vivo* γHV control, we focussed on the well-characterized OT-I TcR (Figure 3).

We first infected OT-I mice with MuHV-4 expressing OVA or APLs with comparable H2K^b^ binding (Q4, V4, G4, R4), and measured host colonization by infectious center assay of spleens 9 and 11 days later (Figure 3A). vE1 and vA8 were not utilized since they bind MHC class I less efficiently precluding analysis of T cell functional avidity because target concentrations are different. There was a clear correlation between CTL functional avidity (Figure 1C) and *in vivo* virus control. The antagonist epitope (R4) allowed no control - titers were equivalent to those of the epitope-negative vWT; the others showed a hierarchy of control (OVA>Q4>V4>G4) that matched exactly their hierarchy of functional avidity (and not their minor differences in H2K^b^ binding). Low titers of pre-formed infectious virus were found in some mice, but generally in proportion to their latent titers, consistent with reactivation of a fixed fraction of the latent viral load; we saw no evidence that M2-associated epitope presentation created a significant new lytic CTL target.

To confirm that the immune control was by CTL, we treated mice with a depleting, CD8-specific mAb from the time of infection (Figure 3B–D). Each virus then reached equivalent titers to the wild-type. While the depletions were highly effective (Figure 3C), they had little effect on the day 11 spleen titers of vWT (Figure 3D). This result was consistent with previous publications [36], [55] and with the lack of known H2^b^-restricted MuHV-4 latency epitopes. Thus, introducing latent epitope recognition caused new, CD8-dependent virus attenuation in proportion to the functional avidity of that epitope for the dominant TcR.

### CTL functional avidity in the context of normalized T cell repertoire

**Figure 4:**
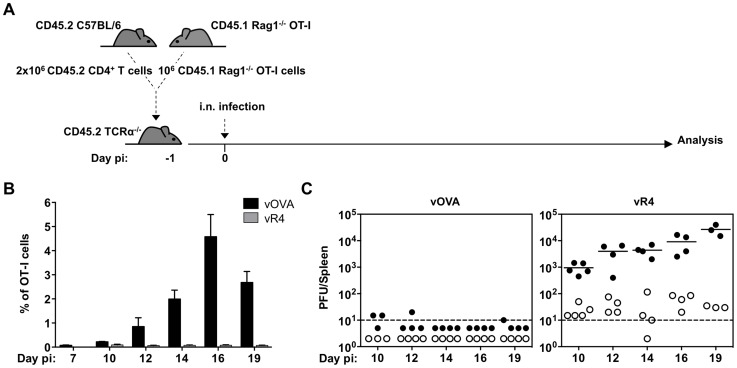
vOVA infection of TCRα^−/−^ mice reconstituted with CD4^+^/OT-I T cells elicits a strong OT-I response and suppression of splenic colonization. CD4^+^ T cells from C57BL/6 lymph nodes and OT-I T cells from CD45.1 Rag-1^−/−^ OT-I lymph nodes were intravenously transferred to TcRα^−/−^ mice one day prior to infection with vOVA or vR4 (10^3^ PFU). (A) Schematic diagram of the experimental setting. (B) Kinetics of *in vivo* OT-I CTL expansion in spleens of mice infected with vOVA (black bars) or vR4 (grey bars) determined by FACS staining of CD45.1^+^CD8α^+^ cells (arithmetic mean +/− SEM). (C) Latent infection in spleens was quantified by explant co-culture assay (closed circles) and pre-formed infectious virus by plaque assay (open circles). Each circle shows the titre of 1 mouse. Horizontal bars show arithmetic means. The dashed line shows the limit of detection of the assay.

OT-I mice provided a useful starting point for *in vivo* analysis of single TcR function. However their limited CD4^+^ T cell repertoire impairs GC formation and so the ability of MuHV-4 to drive B cell proliferation. Hence, to define the impact of TcR functional avidity in an environment more conducive to lymphoproliferation, we adoptively transferred lymphocytes from Rag-1^−/−^OT-I mice and purified CD4^+^ T cells from C57BL/6 mice into TcRα^−/−^ recipients (Figure 4A). Thus the reconstituted mice had polyclonal CD4^+^ T cells and a TcRαβ^+^CD8^+^ T cell compartment of modest size that was restricted to OT-I cells. (Most CD8^+^ T cells of TcRα^−/−^ mice are TcRγδ^+^TcRαβ^−^.) Infecting these with vWT led to a robust proliferation of infected GC B cells (Figure S2 and S3). Infecting them with vOVA elicited a strong OT-I response (Figure 4B) and suppression of splenic colonization (Figure 4C); by contrast vR4, which expressed an antagonist epitope, elicited no OT-I response and reached high titers (Figure 4C). Therefore these mice provided a new and informative window onto how TcR engagement by a latency epitope affects virus-driven lymphoproliferation.

### Sub-optimal CTL functional avidity still allows control of virus-driven lymphoproliferation

**Figure 5:**
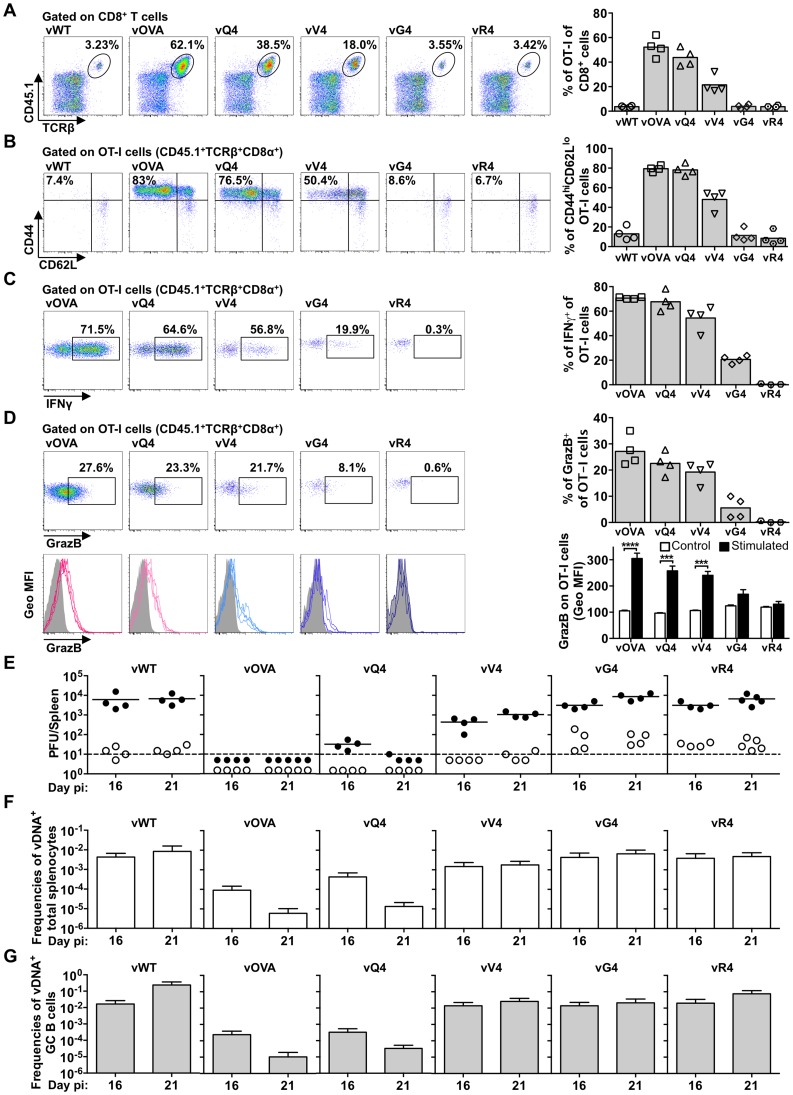
Suboptimal CTL functional avidity still allows control of virus-driven lymphoproliferation. Reconstituted TcRα^−/−^ mice (described in Figure 4A). were i.n. infected. (A–D) At 16 days the frequency, phenotype and effector function of transferred OT-I T cells was analyzed by flow cytometry. (A) Representative FACS plots from individual animals show the frequency of OT-I (CD45.1^+^TcRβ^+^CD8α^+^) cells within total CD8^+^ T cells. vOVA, vQ4 and vV4 induced significant expansion of OT-I cells in comparison with vWT (p<0.0001, p<0.0001, p = 0.002, respectively; by ordinary one-way ANOVA followed by Tukey's multiple comparisons test). vWT, vG4 and vR4 did not significantly increase OT-I cell numbers (p>0.9). (B) The activation phenotype of OT-I cells was determined by staining he CD45.1^+^TcRβ^+^CD8α^+^ population for CD44 and CD62L. vOVA, vQ4 and vV4 induced significantly more OT-I cell activation than vWT (p<0.0001); vG4 and vR4 were not significantly different from vWT (p>0.9). (C–D) The effector function of OT-I cells was determined as % CD45.1^+^TcRβ^+^CD8α^+^ cells producing (C) IFN-γ and (D) granzyme B by intracellular cytokine staining following *ex vivo* stimulation with OVA or the corresponding APL peptide. Histograms show geometric mean fluorescence intensities of granzyme B staining relative to an antibody isotype control (shaded area). Representative FACS plots from individual animals (left panels) and compiled percentages (right panels) are shown. Each point shows 1 mouse; 4 mice were analyzed per group; the bars shows means. *** p<0.001, **** p<0.0001; using Student's 2-tailed unpaired t-test. (E) At 16 and 21 days, spleens were titrated for latent virus (closed circles) and infectious virus (open circles). Each circle shows the titre of 1 mouse and the horizontal bars show means. The dashed line shows the limit of detection of the assay. At 16 and 21 days vOVA, vQ4 and vV4 showed significantly lower latent loads than vWT (d16: vOVA p = 0.02, vQ4 p = 0.02, vV4 p = 0.03; d21: vOVA p = 0.004, vQ4 p = 0.006, vV4 p = 0.02; by ordinary one-way ANOVA and Dunnett's multiple comparisons test). Latent loads of vG4 and vR4 were not significantly different from vWT (d16: vG4 p = 0.4, vR4 p = 0.4; d21: vG4 p = 0.8, vR4 p = 1.0). (F–G) Reciprocal frequencies of viral DNA^+^ cells in (F) total splenocytes and (G) purified GC B cells. Bars show frequencies of viral DNA-positive cells with 95% confidence intervals.

We then infected reconstituted mice with MuHV-4 expressing OVA or APLs (Figure 5). At day 16 post-infection OT-I T cell expansion was greatest for vOVA, reduced for vQ4, reduced further for vV4, and close to background for vG4 and vR4 (Figure 5A). Thus it correlated well with the epitope functional avidity measured in Figure 1C (OVA>Q4>V4>G4>R4). Specifically, the 14-fold avidity reduction of Q4 only modestly reduced CTL cell expansion, and the 4000-fold reduction of V4 caused further reduction but still did not ablate it entirely. The CTL response declined to background only when the avidity was reduced 200,000-fold (G4). Therefore the immune response showed a surprisingly large tolerance for sub-optimal TcR engagement.

Similar results were obtained for OT-I T cell activation (loss of CD62L, Figure 5B). We analyzed CTL function further by intracellular staining for IFN-γ (Figure 5C) and Granzyme B (Figure 5D) after *ex vivo* stimulation with the corresponding peptide epitope. The responses to vG4 and vR4 were hard to assess due to low CTL numbers; but those to vQ4 and vV4 showed comparable functionality to vOVA. (Note that the peptide concentration used was only just sufficient for maximal stimulation by V4 in Figure 1C) Therefore there was no sign of vQ4 and vV4 eliciting CTL responses that were functionally impaired (or functionally enhanced); they simply elicited responses that were smaller.

Virus titers (Figure 5E) were reduced markedly by OVA expression, only marginally less by Q4, and not significantly by G4 or R4. V4 expression gave an intermediate phenotype, with titers significantly below those of the vWT control and significantly above those of vOVA. The frequencies of viral DNA^+^ cells in spleens (Figure 5F and Table S1) showed a similar hierarchy (vWT = vG4 = vR4>vV4>vQ4>vOVA). The viral DNA^+^ frequencies of flow cytometrically sorted GC B cells (Figure 5G and Table 3) showed less discrimination. Nonetheless the trends were similar, and these results were further corroborated by analysis of YFP expression in GC B cells (Figure S4). Therefore high functional avidity (vOVA) gave marked CTL expansion and low virus titers; a 14-fold avidity reduction (vQ4) have remarkably similar results; a 200,000-fold avidity reduction abolished virus control (vG4); and a 4000-fold reduction gave an intermediate phenotype (vV4). OT-I TcR engagement by M2-derived OVA was therefore considerably above the threshold required for *in vivo* viral control, and low functional avidity compromised viral control via reduced CTL expansion, rather than by differentially affecting CTL effector function.

**Table 3:** Reciprocal frequency of MuHV-4 infection in GC B cells^a^ of reconstituted TCRα^−/−^ mice.

Virus	Day p.i.	Reciprocal frequency^b^ of viral DNA^+^ cells (95% CI)		% Cells^c^	% Purity^d^
vWT	16	61	(38–158)	3.13	97.3
	21	4	(3–9)	6.36	97.4
vOVA	16	41,748	(25,873–108,104)	1.95	97.0
	21	id	>96,432^e^	4.88	98.4
vQ4	16	3,042	(1,874–8,064)	3.50	97.0
	21	29,920	(19,237–67,294)	4.87	97.0
vV4	16	72	(45–176)	3.00	98.2
	21	39	(25–84)	8.83	99.0
vG4	16	72	(45–176)	3.08	97.0
	21	32	(18–108)	6.68	98.0
vR4	16	50	(29–167)	2.46	97.4
	21	16	(9–53)	7.99	97.0

a Data were obtained from pools of 4 to 5 spleens.

b Frequencies were calculated by limiting-dilution analysis with 95% confidence intervals (CI).

c The percentage of GC B cells from total spleen was estimated by FACS analysis.

d The purity of sorted cells was determined by FACS analysis.

e Estimated based upon less than 3 different dilution sets.

id; indeterminable.

## Discussion

Gamma-herpesvirus epitope recognition by CTL has been studied extensively [1], [54], but ours is the first quantitative assessment of how epitope/MHC class I/TcR complex formation affects host colonization. Where no latency epitope expression existed, introducing one led to a profound, CTL-dependent suppression of virus-driven lymphoproliferation. This was consistent with the impact of endogenous epitope presentation in H2^d^ mice [29]. The latter affected only long-term viral loads; OVA expression in H2^b^ mice also conferred susceptibility to CTL during acute lymphoproliferation, when trans-acting immune evasion operates [1]. This greater effect of epitope presentation possibly reflected differences in host susceptibility to immune evasion: the MuHV-4 K3 degrades H2K^b^ relatively poorly [19] and degrades TAP better in H2^d^ than H2^b^ cells [20].

The precise cellular targets for CD8^+^ T cell recognition of M2-linked epitopes remain unknown. One possibility is proliferating germinal centre B cells, as B cells are a major site of M2 expression [10], [34]. Infected B cells could also be recognized before the onset of proliferation; and as myeloid cells transfer infection to B cells [56], CD8^+^ T cells could also suppress lymphoproliferation indirectly, by targeting infected myeloid cells [1].

A key point for physiologically relevant epitope presentation is that it conforms to normal latent gene expression. Exogenous promoters such as HCMV IE1 show activity independent of endogenous viral gene expression [57] and this can lead to attenuation [58]. Previous analysis of endogenous M2 epitope [29] established its importance for determining the different long-term latent loads of H2^d^ and H2^b^ mice. Here, to identify presentation thresholds, we made use of the well-characterized SIINFEKL epitope, attaching it to a neutral region of M2 (its C-terminus). This allowed the generation of a very well-defined model epitope with the kinetics and copy number of a known endogenous epitope. Epitope presentation varies with MHC class I genotype. C57BL/6 mice have only 2 MHC class I molecules and appear not to recognize an endogenous M2 epitope. In this context, M2-SIINFEKL illustrated the impact of strong epitope presentation, and wild-type M2 (or M2-vA8) that of poor epitope presentation. The SIINFEKL variants covered the range between, and so allowed us to identify functional recognition thresholds.

Small differences (<1.6-fold) in H2K^b^ epitope binding had no obvious impact on *in vivo* CTL efficacy, but a 60-fold reduction abolished protection and a 6-fold reduction showed a partial phenotype. Thus, M2-linked epitope presentation left little room for sub-optimal MHC class I binding. By contrast when H2K^b^ binding was maintained, reducing TcR functional avidity 14-fold had little effect, reducing it 200,000-fold abolished control, and reducing it 4,000-fold gave an intermediate phenotype. Therefore this aspect of recognition was more flexible even for monoclonal, Rag-1^−/−^ CTL, and a polyclonal population could attack any epitope so long as its MHC class I binding was strong.

In complex viral infections, larger CTL responses are not necessarily more effective responses. These parameters can correlate: MuHV-4 lacking its K3 evasion gene elicits more CTL and achieves lower titers [22]; and our reconstituted mice showed a correlation between more CTL and less virus. But as with latent epitope presentation downstream of ORF73 [11], OVA-specific CTL responses that completely suppressed lymphoproliferation were small compared to lytic epitope responses [51]; and mice infected with vE1 made large epitope-specific responses yet showed poor virus control. We hypothesize that CTL can be stimulated by the key, self-renewing population of infected B cells, when infection is suppressed, but also by infected cells less important to host colonization, when large responses may achieve little. Crucially, viral evasion may make the self-renewing population harder to target. Thus, vE1 showed a strong acute reduction in total viral DNA^+^ cell frequencies, but relative sparing of GC B cells and consequently high long-term virus loads. A position 1 mutation also impairs the control by Rag-1^−/−^OT-I mice of MuHV-4 expressing OVA from an HCMV IE1 promoter [59]. However such mice lack B cells or CD4^+^ T cells, and without CD4^+^ T cells MuHV-4 causes a lethal, chronic lytic infection even with a strong, polyclonal CTL response [60], [61]. Our reconstituted mice maintained both virus-driven lymphoproliferation and infection control without outgrowth of CTL escape mutants. Thus we could relate directly quantitative changes in epitope recognition to the control of lymphoproliferation.

An important task with EBV is to predict *in vivo* CTL efficacy. Extrapolating from CTL numbers and *in vitro* assays alone is clearly problematic. For example, large responses to lytic epitopes in infectious mononucleosis [54] could be interpreted as important, or simply as poor latency epitope recognition when better recognition might preclude large lytic responses and avoid symptoms. The precise relatedness of EBV memory B cell colonization via GCs to MuHV-4 memory B cell colonization via GCs is unknown. But all γHVs have evolved to colonize lymphocytes with maximal efficiency, within limits set ultimately by the immune system, so similar quantitative thresholds would not be surprising. Our data therefore have important general implications for γHV-specific CTL function, and for predicting *in vivo* CTL efficacy from biochemical measures.

## Materials and Methods

### Ethics statement

The study accorded with the Portuguese official Veterinary Directorate (Portaria 1005/92), European Guideline 86/609/EEC, and Federation of European Laboratory Animal Science Associations guidelines on laboratory animal welfare. It was approved by the Portuguese official veterinary department for welfare licensing (protocol AEC_2010_017_PS_Rdt_General) and by the IMM Animal Ethics Committee.

### Mice

CD45.1 C57BL/6, OT-I, Rag-1^−/−^ and TcRα^−/−^ mice were obtained from Jackson Laboratories. CD45.1 Rag-1^−/−^ OT-I mice were obtained by breeding OT-I onto a CD45.1 Rag-1^−/−^ background. C57BL/6 and BALB/c mice were purchased from Charles River Laboratories. All mice were housed under specific pathogen-free conditions at the Instituto de Medicina Molecular and used when 6–12 weeks old. For adoptive transfers to TcRα^−/−^ mice, CD4^+^ T cells were purified by negative selection from pooled lymph nodes of naïve C57BL/6 mice using the CD4^+^ T cell isolation kit (Miltenyi Biotech). OT-I T cells were obtained from pooled lymph nodes of naïve CD45.1 Rag-1^−/−^ OT-I mice. 2×10^6^ CD4^+^ T cells and 10^6^ CD45.1 Rag-1^−/−^ OT-I T cells were adoptively transferred to TcRα^−/−^ recipients via tail vein injection one day prior to infection.

### Generation of recombinant viruses

MuHV-4 recombinants were generated from BAC-cloned viral genomes [29]. OVA and APL epitopes were introduced by PCR at the M2 C-terminus. Briefly, the M2 downstream region (genomic co-ordinates 3846-4029) containing a *Hind*III restriction site followed by the epitope coding region and a stop codon were PCR amplified (Table S2) to attach each epitope to the M2 C-terminus. The PCR products were inserted downstream of a *Hin*DIII/*Xho*I MuHV-4 genomic fragment (nt 4029–5362) in pSP72 (Promega), using a genomic *Bgl*II site (nt 3846) and the engineered *Hin*DIII (nt 4029) restriction site. The constructs were then subcloned into a *Hin*DIII-E MuHV-4 genomic fragment in the pST76K-SR shuttle plasmid, using genomic *Bln*I (nt 3908) and *Xho*I (nt 5362) restriction sites. All PCR-derived regions were sequenced to confirm the integrity of the introduced epitopes and the M2 flanking region. Each recombinant *Hin*DIII-E shuttle plasmid was transformed into *E.coli* carrying the wild type MuHV-4 BAC (pHA3) or a YFP^+^ BAC [50] obtained from Dr Samuel Speck (Emory Vaccine Center, Atlanta). Following multi-step selection, recombinant BAC clones were identified by restriction digestion with *Hin*DIII. The integrity of each BAC was confirmed by digestion with *Bam*HI and *Eco*RI. All viruses were reconstituted by transfecting BAC DNA into BHK-21 cells using FuGENE 6 or X-tremeGENE HP (Roche Applied Science). The *lox*P-flanked BAC cassette was then removed by viral passage through NIH-3T3-CRE cells and limiting dilution cloning. The integrity of each reconstituted virus was checked by PCR of viral DNA across the *Hin*DIII-E region and DNA sequencing across M2.

### Cell culture and viruses

Murine RMA/S cells were cultured in RPMI 1640 with 10% fetal calf serum, 2 mM glutamine and 100 U/ml penicillin and 100 µg/ml streptomycin. NIH-3T3 (ATCC)-CRE cells [22] were grown in Dulbecco's modified Eagle's medium (DMEM) with 10% fetal calf serum, 2 mM glutamine, 100 U/ml penicillin and 100 µg/ml streptomycin. Baby hamster kidney fibroblast cells (BHK-21, ATCC) were cultured in Glasgow's modified Eagle's medium (GMEM) supplemented as above plus 10% tryptose phosphate broth. To prepare viral stocks, low multiplicity infections (0.001 PFU per cell) of NIH-3T3-CRE or BHK-21 cells were harvested after 4 days and titrated by plaque assay [29].

### H2K^b^ stabilization assay and OVA/APLs stimulatory potency

H2K^b^ stabilization was determined with TAP-deficient RMA/S cells. These were incubated overnight at 26°C to promote the export of empty H2K^b^ complexes, then loaded with graded concentrations of OVA or APL peptides (Thermo Scientific) for 2 h at 26°C and subsequently transferred to 37°C for 2 h to destabilize empty MHC molecules [43]. The cells were then washed twice, stained with anti-H2K^b^ (AF6-88.5.5.3, eBioscience), and analysed on a LSR Fortessa (BD Biosciences). Mean fluorescence intensities were determined with FlowJo (Tree Star). To measure the *ex vivo* stimulation of naïve OT-I T cells by OVA and APLs, CD8^+^ T cells from the spleens of naïve OT-I mice were purified by negative selection (CD8^+^ T cell isolation kit, Miltenyi Biotech); for equivalent peptide/MHC class I numbers, irradiated (7500 rads) RMA/S cells were loaded with different peptides at 26°C, then incubated at 37°C; and 5×10^4^ OT-I T cells were cultured with 2.5×10^4^ RMA/S cells for 72 h at 37°C. IFNγ levels in culture supernatants were measured by ELISA (DuoSet ELISA development kit, R&D Systems). The data were fitted to sigmoidal dose-response curves and EC_50_ values calculated using GraphPad Prism.

### *In vivo* infections and virus assays

Groups of 6- to 8-week old BALB/c and C57BL/6 mice were inoculated i.n. with 10^4^ PFU of MuHV-4. 8- to 12-week old OT-I and TCRα^−/−^ mice were inoculated i.n. with 10^3^ PFU of MuHV-4. All virus inoculations were in 20 µl of PBS under isofluorane anaesthesia. At different days post-infection lungs or spleens were removed and processed for subsequent analysis. Titres of infectious virus were determined by plaque assay of freeze-thawed lung or spleen homogenates using BHK-21 cells. Latent virus loads were quantified by explant co-culture of splenocytes with BHK-21 cells. Plates were incubated for 4 (plaque assay) or 5 (explant co-culture assay) days, then fixed with 4% formaldehyde and stained with 0.1% toluidine blue. Viral plaques were counted with a plate microscope. The frequency of MuHV-4 genome-positive cells was determined by limiting dilution combined with real time PCR [10]. Splenocytes were pooled from 4–5 mice. GC B cells (CD19^+^CD95^hi^GL7^hi^) were purified from pools of 4 or 5 spleens using a BD FACSAria Flow Cytometer (BD Biosciences). Cells were serially two-fold diluted and eight replicates of each dilution were analysed by real time PCR (Rotor Gene 6000, Corbett Life Science). The primer/probe sets were specific for the MuHV-4 M9 gene (5′ primer: GCCACGGTGGCCCTCTA; 3′ primer: CAGGCCTCCCTCCCTTTG; probe: 6-FAM-CTTCTGTTGATCTTCC-MGB). Samples were subjected to a melting step of 95°C for 10 min followed by 40 cycles of 15 s at 95°C and 1 min at 60°C. Real-time PCR data was analysed on the Rotor Gene 6000 software. The purity of sorted populations was always >96%. *In situ* hybridization with a digoxigenin-labelled riboprobe encompassing MuHV-4 vtRNAs 1–4 and microRNAs 1–6 was performed on formalin-fixed, paraffin-embedded spleen sections [29], using probes generated by T7 transcription of pEH1.4.

### *In vivo* cytotoxicity assay

Splenocytes from naïve CD45.1 C57BL/6 mice were used as targets and controls. Targets were pulsed with 1 µM OVA, E1 or A8 peptides for 1 h at 37°C, then labeled with 1 µM carboxyfluorescein succinimidyl ester (CFSE) (Molecular Probes). Controls were left unpulsed and labeled with 0.1 µM CFSE. Cells were washed three times then injected intravenously as a 50∶50 mix of CFSE^hi^ and CFSE^lo^ cells (4×10^6^) into mice infected with vWT, vOVA, vE1 or vA8. The same mixes were injected intravenously into vWT infected C57BL/6 controls to ensure equal transfer. On the next day splenocytes were harvested and the proportion of CFSE^hi^ and CFSE^lo^ cells among CD45.1 splenocytes was analysed by FACS. Target cell killing was calculated as (% CFSE^lo^/% CFSE^hi^), with % = 100−(ratio in vWT infected/ratio in vOVA, vE1 or vA8 infected)×100.

### CD8^+^ T cell depletions

MuHV-4 infected OT-I mice were depleted of CD8^+^ T cells by 5 intraperitoneal injections of 200 µg monoclonal antibody YTS 169.4. Splenocytes from control or depleted mice were stained with anti-CD8α (53-6.7) (BD Pharmingen) and analysed on a LSR Fortessa (BD Biosciences).

### *Ex vivo* stimulation and intracellular cytokine staining

Splenocytes (2×10^6^) from infected mice were stimulated for 5 h at 37°C with 10 µg/ml peptide (OVA, APLs or VSV NP_52-59_) in RPMI 1640/10% fetal calf serum/2 mM glutamine/100 U/ml penicillin/100 µg/ml streptomycin/50 µM 2-mercaptoethanol/10 U/ml recombinant murine IL-2 (PeproTech)/10 µg/ml Brefeldin A. Cells were then washed, blocked with anti-CD16/32 (2.4G2) (BD Pharmingen), surface stained with anti-CD8α ± anti-CD45.1 (for OT-I T cells), fixed and permeabilized with Foxp3 staining buffer (eBioscience) and stained with anti-IFNγ (XMG1.2) (BD Pharmingen), anti-Granzyme B (NGZB) or anti-IgG2ak Isotype control (eBioscience). Samples were analysed on a LSR Fortessa (BD Biosciences).

### Flow cytometry

Splenocytes were treated with red blood cell lysis buffer, blocked with anti-CD16/32 (2.4G2, BD Pharmingen, 10 min), and stained at 4°C in PBS/2% FCS 30 minutes: anti-CD95 (Jo2), anti-CD19 (1D3), anti-CD8α (53-6.7), anti-IFNγ (XMG1.2) (BD Pharmingen); anti-CD45.1 (A20), anti-CD45.2 (104), anti-CD44 (IM7), anti-CD62L (MEL-14) (Biolegend); anti-GL7 (GL7), anti-H2K^b^ (AF6-88.5.5.3), anti-TCRβ (H57-597), anti-GranzymeB (NGZB), anti-IgG2ak Iso control (eBR2a) (eBioscience). For biotinylated antibodies, an additional 20 minutes incubation with streptavidin was performed. MuHV-4 infected cells were identified by YFP expression. H2K^b^ tetramers conjugated to PE were a kind gift from Dr Hidde L. Ploegh (Whitehead Institute for Biomedical Research, Massachusetts Institute of Technology, Cambridge). Conditional ligand was exchanged for SIINFEKL (OVA), SIIQFEKL (Q4), SIIVFEKL (V4), SIIGFEKL (G4), SIIRFEKL (R4), EIINFEKL (E1) or RGYVYQGL (VSV NP_52-59_) peptides (Thermo Scientific). Streptavidin-APC or -PerCP (BD Pharmingen) was used to reveal biotinylated antibodies. Samples were acquired on a LSR Fortessa using DIVA (BD Biosciences) and analysed with FlowJo (Tree Star, Inc.).

### Statistical analysis

Data comparisons between groups were performed by an unpaired two-tailed t-test or ordinary one-way ANOVA as appropriate. Mean +/− SEM and statistics were calculated with GraphPad Prism Software. For limiting dilution analysis 95% confidence intervals were determined as described [10].

### PCR primers

Primers used for attaching each epitope to MuHV-4 M2 C-terminus are detailed in supplemental Table S2.

## Supporting Information

Figure S1 **Characterization of MuHV-4 YFP recombinants expressing OVA or APLs linked to M2.** (A) PCR analysis of recombinant viral DNA to confirm genome integrity in the HinDIII-E region. High molecular weight DNA was purified from lytically infected BHK-21 cells. A schematic representation of the MuHV-4 genome, amplicon genomic coordinates and expected size for each PCR product are shown. (B) Latent infection in spleens of intranasally infected (10^4^ PFU) BALB/c (H2^d^) mice was quantified by explant co-culture assay (closed symbols) at day 14 post-infection. Pre-formed infectious virus was measured by plaque assay (open symbols). Latent loads of MuHV-4 YFP recombinants expressing OVA or APLs were not significantly different from MuHV-4 YFP (vWT) (p>0.05, by ordinary one-way ANOVA followed by Dunnett's multiple comparisons test). Each point shows the titre of 1 mouse, horizontal lines indicate arithmetic means and the dashed horizontal line the limit of detection of the assay. Data were reproduced in two independent experiments. (C) Phenotype of infected cells (YFP expressing cells) was analysed by FACS, by overlapping GC (CD19^+^CD95^hi^GL7^hi^) B cells and YFP^+^ B cells FACS plots. Representative FACS plots from individual animals are shown. Five animals were analysed per group and data were reproduced in two independent experiments.
(TIF)

Figure S2 **Reconstitution of TCRα^−/−^ mice with CD4^+^ T cells leads to robust GC reactions upon MuHV-4 infection.** 2×10^6^ CD4^+^ T cells purified from pooled lymph nodes of naïve C57BL/6 mice were intravenously transferred into age and sex matched TCRα^−/−^ mice one day prior to infection with 10^3^ PFU of MuHV-4 YFP (vWT). At 14 days post-infection mice were sacrificed, spleens were dissected and single splenocyte suspensions were stained for GC B cells and analysed by FACS. (A) Schematic diagram of the experimental setting. (B) Representative FACS plots show the frequency of GC (CD19^+^CD95^hi^GL7^hi^) B cells in spleens of the following experimental controls: non-transferred naïve TCRα^−/−^ mice, CD4-transferred naïve TCRα^−/−^ mice, non-transferred TCRα^−/−^ mice infected with vWT, CD4-transferred TCRα^−/−^ mice infected with vWT, and CD4 and OT-I T cells co-transferred TCRα^−/−^ mice infected with vWT. Four mice were analysed per group and data were reproduced in two independent experiments.
(TIF)

Figure S3 **TCRα^−/−^ mice reconstituted with CD4^+^ and OT-I T cells show robust proliferation of MuHV-4 infected GC B cells.** CD4^+^ T cells from C57BL/6 lymph nodes and OT-I T cells from CD45.1 Rag-1^−/−^ OT-I mice lymph nodes were intravenously transferred to TCRα^−/−^ mice 1 day prior to infection with MuHV-4 YFP (10^3^ PFU). (A) Schematic diagram of the experimental setting. (B) Frequencies of GC (CD19^+^CD95^hi^GL7^hi^) B cells. (C) Frequency of YFP^+^ cells in GC B cells. (D) Phenotype of infected cells analyzed by overlapping GC B cells and YFP^+^ B cells FACS plots. Representative FACS plots from individual animals are shown (top panels) and compiled percentages are presented in the graphics below. Each point represents an individual mouse; grey bars indicate the average percentage.
(TIF)

Figure S4 **YFP expression in GC B cells of reconstituted TCRα^−/−^ mice infected with MuHV-4 recombinants expressing OVA or APLs.** TCRα^−/−^ mice were adoptively transferred with polyclonal CD4^+^ T cells and CD45.1 Rag1^−/−^ OT-I cells one day prior to infection (10^3^ PFU) with MuHV-4 YFP (vWT) or MuHV-4 YFP expressing the indicated epitopes. At 16 (A and B) and 21 (C and D) days post-infection spleens were removed and analysed by FACS. (A and C) Frequencies of GC (CD19^+^CD95^hi^GL7^hi^) B cells. (B and D) Frequency of YFP^+^ cells in GC B cells. FACS plots show data obtained from pools of 4 or 5 spleens per group of animals.
(TIF)

Table S1 **Reciprocal frequency of MuHV-4 infection in total splenocytes^a^ of reconstituted TCRα^−/−^ mice.**
(DOC)

Table S2 **Primers used for attaching each epitope to MuHV-4 M2 C-terminus.**
(DOC)
