# Supplementary figures and images for: Defining Immune Engagement Thresholds for In Vivo Control of Virus-Driven Lymphoproliferation
Source: PLoS Pathog. 2014 Jun 26;10(6):e1004220. doi: 10.1371/journal.ppat.1004220 (PMC4072806; doi:10.1371/journal.ppat.1004220)

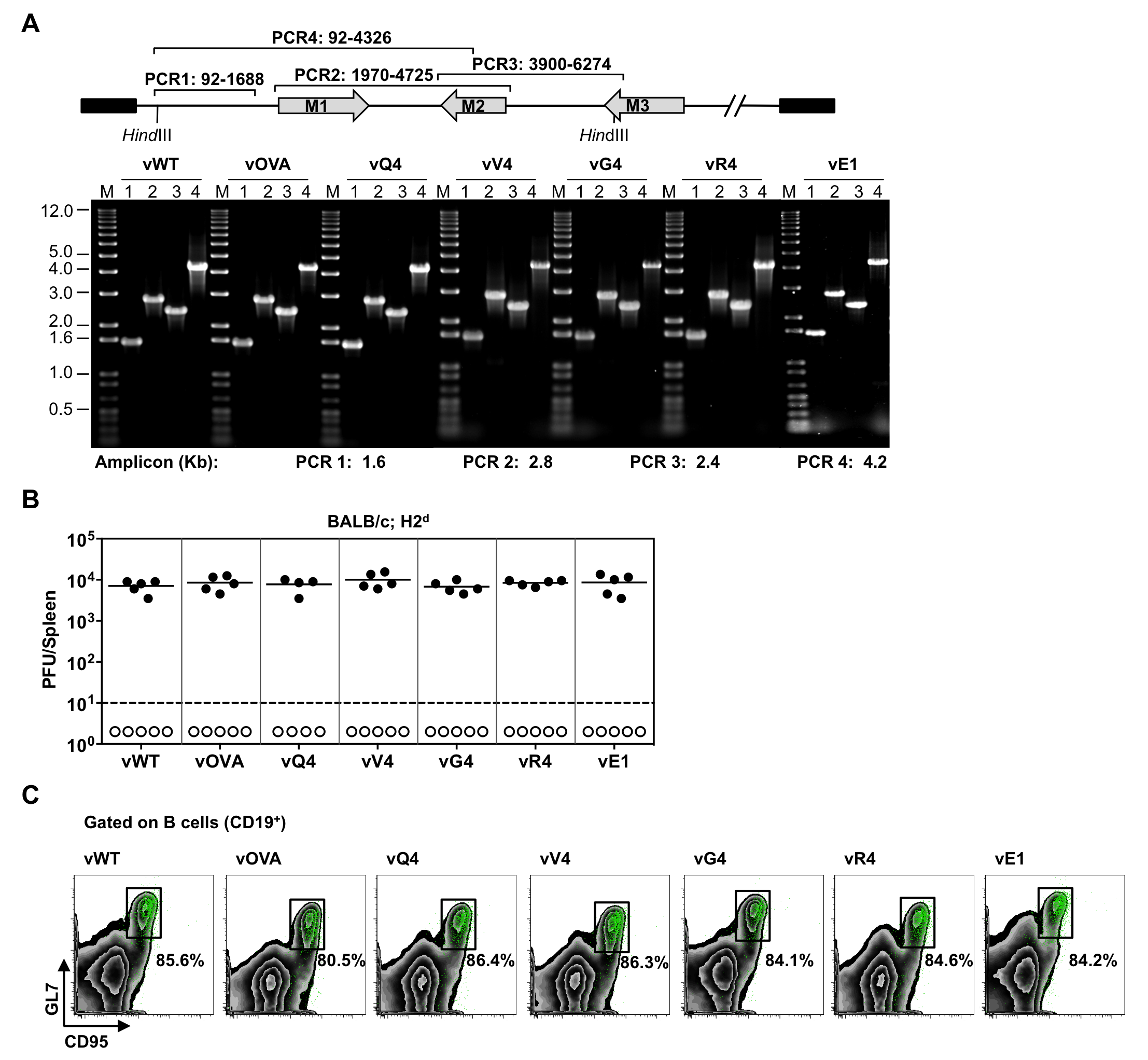

Supplement: Figure S1 — Characterization of MuHV-4 YFP recombinants expressing OVA or APLs linked to M2. (A) PCR analysis of recombinant viral DNA to confirm genome integrity in the HinDIII-E region. High molecular weight DNA was purified from lytically infected BHK-21 cells. A schematic representation of the MuHV-4 genome, amplicon genomic coordinates and expected size for each PCR product are shown. (B) Latent infection in spleens of intranasally infected (104 PFU) BALB/c (H2d) mice was quantified by explant co-culture assay (closed symbols) at day 14 post-infection. Pre-formed infectious virus was measured by plaque assay (open symbols). Latent loads of MuHV-4 YFP recombinants expressing OVA or APLs were not significantly different from MuHV-4 YFP (vWT) (p>0.05, by ordinary one-way ANOVA followed by Dunnett's multiple comparisons test). Each point shows the titre of 1 mouse, horizontal lines indicate arithmetic means and the dashed horizontal line the limit of detection of the assay. Data were reproduced in two independent experiments. (C) Phenotype of infected cells (YFP expressing cells) was analysed by FACS, by overlapping GC (CD19+CD95hiGL7hi) B cells and YFP+ B cells FACS plots. Representative FACS plots from individual animals are shown. Five animals were analysed per group and data were reproduced in two independent experiments. (TIF) [file ppat.1004220.s001.tif]

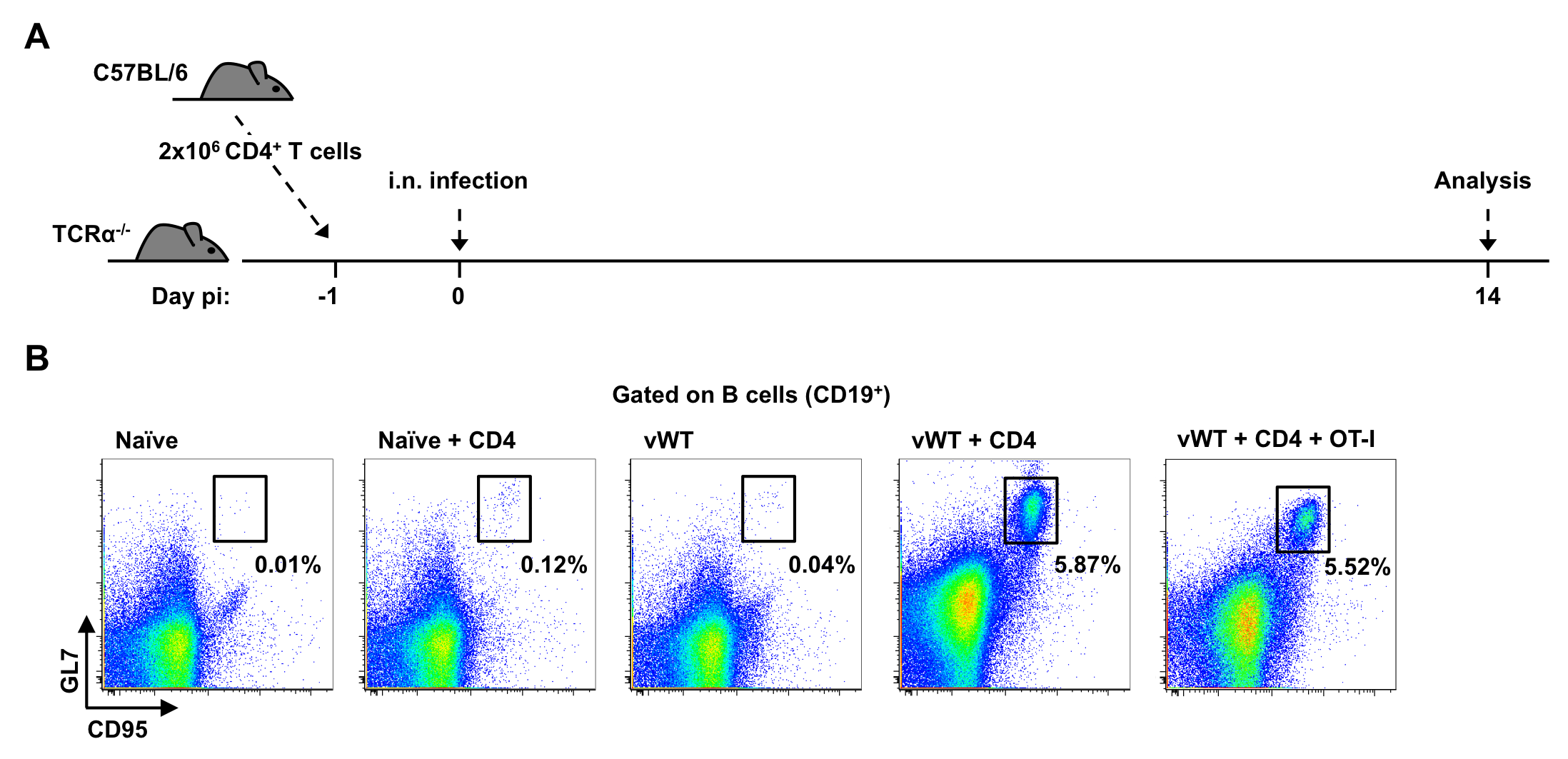

Supplement: Figure S2 — Reconstitution of TCRα−/− mice with CD4+ T cells leads to robust GC reactions upon MuHV-4 infection. 2×10^6^ CD4+ T cells purified from pooled lymph nodes of naïve C57BL/6 mice were intravenously transferred into age and sex matched TCRα−/− mice one day prior to infection with 103 PFU of MuHV-4 YFP (vWT). At 14 days post-infection mice were sacrificed, spleens were dissected and single splenocyte suspensions were stained for GC B cells and analysed by FACS. (A) Schematic diagram of the experimental setting. (B) Representative FACS plots show the frequency of GC (CD19+CD95hiGL7hi) B cells in spleens of the following experimental controls: non-transferred naïve TCRα−/− mice, CD4-transferred naïve TCRα−/− mice, non-transferred TCRα−/− mice infected with vWT, CD4-transferred TCRα−/− mice infected with vWT, and CD4 and OT-I T cells co-transferred TCRα−/− mice infected with vWT. Four mice were analysed per group and data were reproduced in two independent experiments. (TIF) [file ppat.1004220.s002.tif]

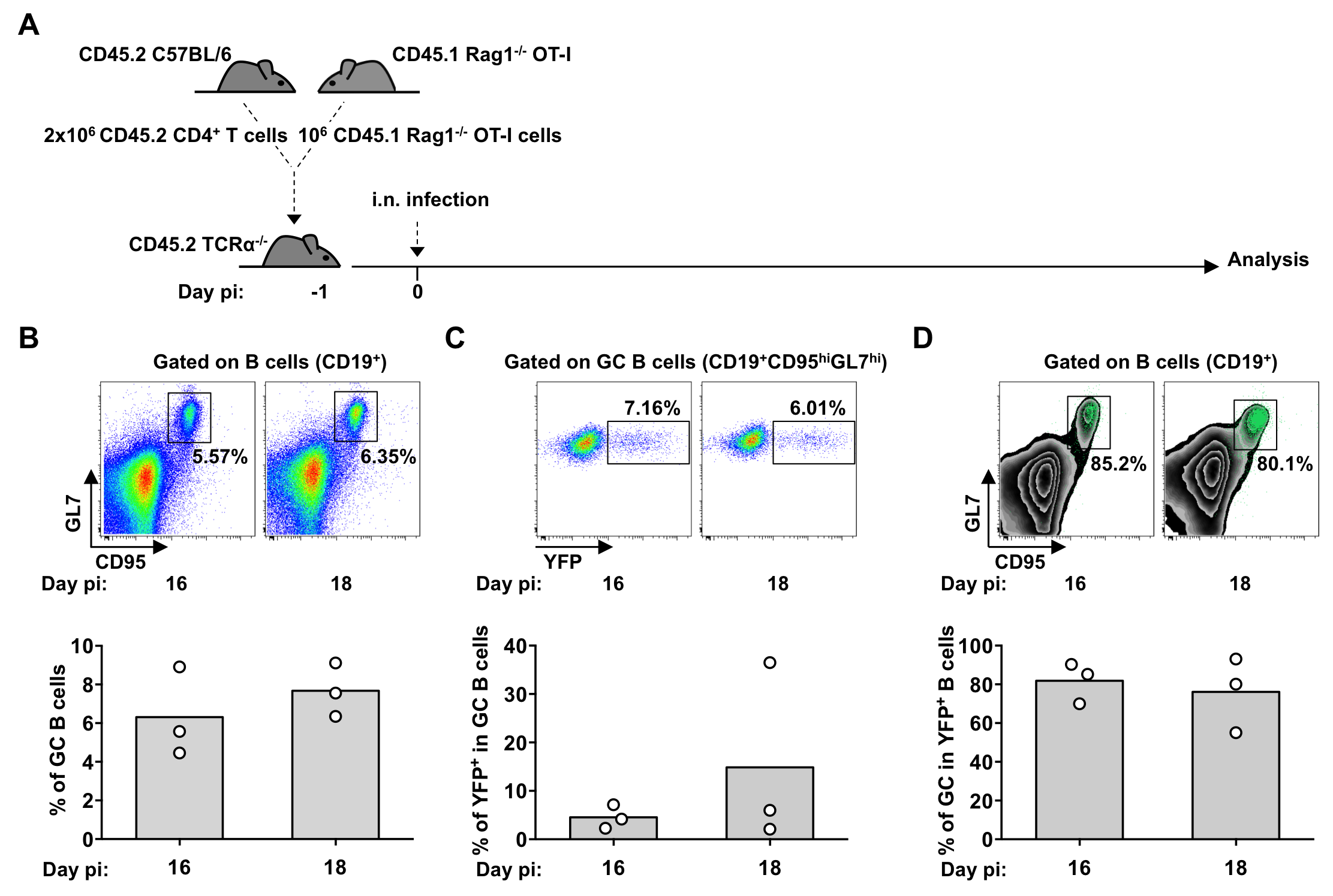

Supplement: Figure S3 — TCRα−/− mice reconstituted with CD4+ and OT-I T cells show robust proliferation of MuHV-4 infected GC B cells. CD4+ T cells from C57BL/6 lymph nodes and OT-I T cells from CD45.1 Rag-1−/− OT-I mice lymph nodes were intravenously transferred to TCRα−/− mice 1 day prior to infection with MuHV-4 YFP (103 PFU). (A) Schematic diagram of the experimental setting. (B) Frequencies of GC (CD19+CD95hiGL7hi) B cells. (C) Frequency of YFP+ cells in GC B cells. (D) Phenotype of infected cells analyzed by overlapping GC B cells and YFP+ B cells FACS plots. Representative FACS plots from individual animals are shown (top panels) and compiled percentages are presented in the graphics below. Each point represents an individual mouse; grey bars indicate the average percentage. (TIF) [file ppat.1004220.s003.tif]

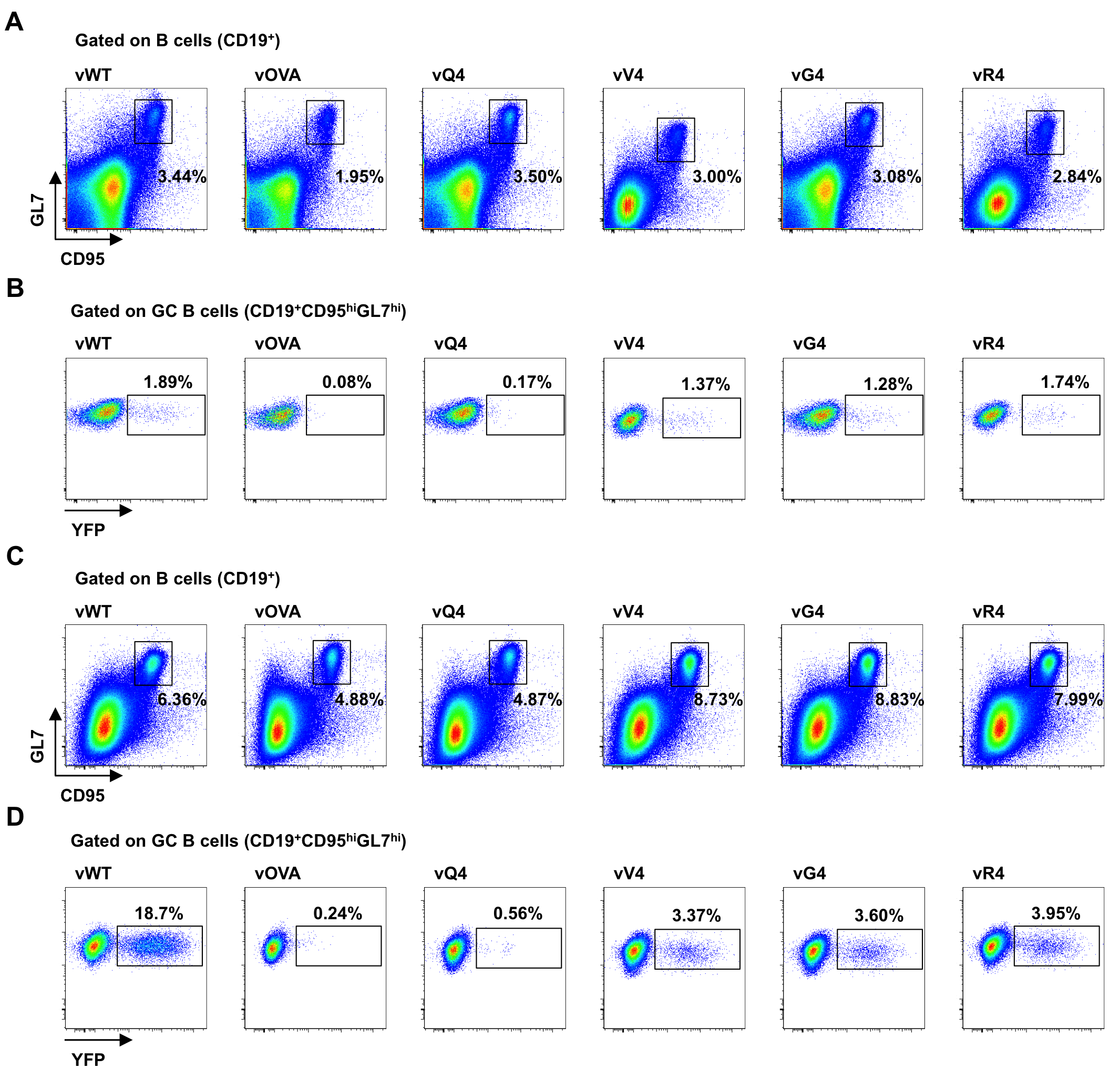

Supplement: Figure S4 — YFP expression in GC B cells of reconstituted TCRα−/− mice infected with MuHV-4 recombinants expressing OVA or APLs. TCRα−/− mice were adoptively transferred with polyclonal CD4+ T cells and CD45.1 Rag1−/− OT-I cells one day prior to infection (103 PFU) with MuHV-4 YFP (vWT) or MuHV-4 YFP expressing the indicated epitopes. At 16 (A and B) and 21 (C and D) days post-infection spleens were removed and analysed by FACS. (A and C) Frequencies of GC (CD19+CD95hiGL7hi) B cells. (B and D) Frequency of YFP+ cells in GC B cells. FACS plots show data obtained from pools of 4 or 5 spleens per group of animals. (TIF) [file ppat.1004220.s004.tif]
