# Supplementary material for: Defining Immune Engagement Thresholds for In Vivo Control of Virus-Driven Lymphoproliferation
Source: PLoS Pathog. 2014 Jun 26;10(6):e1004220. doi: 10.1371/journal.ppat.1004220 (PMC4072806; doi:10.1371/journal.ppat.1004220)
Supplement: Table S1 — Reciprocal frequency of MuHV-4 infection in total splenocytesa of reconstituted TCRα−/− mice. (DOC) [file ppat.1004220.s005.doc]

**Table S1. Reciprocal frequency of MuHV-4 infection in total splenocytes^a^ of reconstituted TCRα^-/-^ mice.**

| **Virus** | **Day p.i.** | **Reciprocal frequency^b^ of viral DNA^+^ cells (95% CI)** | |
| --- | --- | --- | --- |
| vWT | 16 | 232 | (150-509) |
|  | 21 | 119 | (63-991) |
| vOVA | 16 | 11,010 | (6,845-28,119) |
|  | 21 | id | >161,924^c^ |
| vQ4 | 16 | 2,296 | (1,442-5,630) |
|  | 21 | 74,799 | (47,030-182,682) |
| vV4 | 16 | 687 | (427-1,755) |
|  | 21 | 567 | (371-1,201) |
| vG4 | 16 | 231 | (139-688) |
|  | 21 | 152 | (99-331) |
| vR4 | 16 | 257 | (150-870) |
|  | 21 | 211 | (134-491) |

^a^Data were obtained from pools of 4 to 5 spleens.

^b^Frequencies were calculated by limiting-dilution analysis with 95% confidence intervals (CI).

^c^Estimated based upon less than 3 different dilution sets.

id; indeterminable.
