# Supplementary material for: Defining Immune Engagement Thresholds for In Vivo Control of Virus-Driven Lymphoproliferation
Source: PLoS Pathog. 2014 Jun 26;10(6):e1004220. doi: 10.1371/journal.ppat.1004220 (PMC4072806; doi:10.1371/journal.ppat.1004220)
Supplement: Table S2 — Primers used for attaching each epitope to MuHV-4 M2 C-terminus. (DOC) [file ppat.1004220.s006.doc]

**Table S2. Primers used for attaching each epitope to MuHV-4 M2 C-terminus.**

| **MuHV-4 recombinant** | **Primer sequence (5’-3’)** |
| --- | --- |
| **vOVA** | AAAAAGCTT**AGGAGTATAATCAACTTTGAAAAACTGTAA**CAGTGAAGGTGCTAACGCAGAA |
| **vQ4** | AAAAAGCTT**AGGAGTATAATC*CAG*TTTGAAAAACTGTAA**CAGTGAAGGTGCTAACGCAGAA |
| **vV4** | AAAAAGCTT**AGGAGTATAATC*GTG*TTTGAAAAACTGTAA**CAGTGAAGGTGCTAACGCAGAA |
| **vG4** | AAAAAGCTT**AGGAGTATAATC*GGC*TTTGAAAAACTGTAA**CAGTGAAGGTGCTAACGCAGAA |
| **vE1** | AAAAAGCTT**AGG*GAG*ATAATCAACTTTGAAAAACTGTAA**CAGTGAAGGTGCTAACGCAGAA |
| **vR4** | AAAAAGCTT**AGGAGTATAATC*AGG*TTTGAAAAACTGTAA**CAGTGAAGGTGCTAACGCAGAA |
| **vA8** | AAAAAGCTT**AGGAGTATAATCAACTTTGAAAAA*GCC*TAA**CAGTGAAGGTGCTAACGCAGAA |

Engineered *Hin*DIII restriction site is underlined.

Epitope coding region is in bold.

Alterations introduced in native OVA DNA sequence are in bold italics.

Stop codon is underlined and in bold.
